# covRNA: discovering covariate associations in large-scale gene expression data

**DOI:** 10.1186/s13104-020-04946-1

**Published:** 2020-02-24

**Authors:** Lara Urban, Christian W. Remmele, Marcus Dittrich, Roland F. Schwarz, Tobias Müller

**Affiliations:** 1 Department of Bioinformatics, Biocenter, University of Würzburg, Am Hubland, Würzburg, Germany; 2 European Molecular Biology Laboratory, European Bioinformatics Institute, Wellcome Genome Campus, Hinxton, Cambridge, UK; 3 Institute of Human Genetics, University of Würzburg, Am Hubland, Würzburg, Germany; 4 Berlin Institute for Medical Systems Biology, Max Delbrück Center, Berlin, Germany

**Keywords:** Multivariate analysis, Fourthcorner analysis, RLQ analysis, Transcriptomics, High-throughput data, Visualization, Ordination methods, RNA-Seq analysis, Microarray analysis

## Abstract

**Objective:**

The biological interpretation of gene expression measurements is a challenging task. While ordination methods are routinely used to identify clusters of samples or co-expressed genes, these methods do not take sample or gene annotations into account. We aim to provide a tool that allows users of all backgrounds to assess and visualize the intrinsic correlation structure of complex annotated gene expression data and discover the covariates that jointly affect expression patterns.

**Results:**

The Bioconductor package covRNA provides a convenient and fast interface for testing and visualizing complex relationships between sample and gene covariates mediated by gene expression data in an entirely unsupervised setting. The relationships between sample and gene covariates are tested by statistical permutation tests and visualized by ordination. The methods are inspired by the fourthcorner and RLQ analyses used in ecological research for the analysis of species abundance data, that we modified to make them suitable for the distributional characteristics of both, RNA-Seq read counts and microarray intensities, and to provide a high-performance parallelized implementation for the analysis of large-scale gene expression data on multi-core computational systems. CovRNA provides additional modules for unsupervised gene filtering and plotting functions to ensure a smooth and coherent analysis workflow.

## Introduction

The biological interpretation of gene expression measurements and related multivariate datasets is a fundamental yet challenging task in computational biology. Ordination methods like Principal Component Analysis or Correspondence Analysis are routinely used for dimension reduction and visualization to identify clusters of samples or co-expressed genes [1]. These methods do not generally take sample or gene annotations into account. Knowledge-driven approaches such as Gene Ontology Analysis [2] and Gene Set Enrichment Analysis [3] look for differentially regulated sets of genes based on prior information. These methods are powerful but specialized hypothesis-based tools. In functional genomics, it is often desirable to test for associations between extensive categorical and numerical sample and gene covariates. Sample covariates may comprise demographic and clinical data or complex phenotype data derived from imaging. Gene-level covariates often include functional ontology, epigenetic modifications, protein phosphorylation or copy-number state. Methods for the efficient and systematic analysis of the relationship between sample and gene covariates mediated by gene expression are lacking.

## Main text

Here we present covRNA (‘covariates of RNA’), a Bioconductor package [4, 5] providing a convenient and fast interface for testing and visualizing the relationship between sample and gene covariates mediated by gene expression in an entirely unsupervised setting. The methods are inspired by the fourthcorner and RLQ analyses used in ecological research for the analysis of species abundance data [6, 7]. While the scope of these analyses is comparable to knowledge-based approaches like GSEA, their inherently unsupervised and hypothesis-free nature provides a huge advantage if no prior knowledge is available. In addition, while approaches like GSEA are based on parametric distributions like the hypergeometric distribution, the here presented analyses are based on simulated distributions to capture and account for respective dataset-specific data structures and modalities.

The RLQ analysis of the ade4 package [7] has previously been applied for the analysis of microarray data describing the time-course effect of steroids on the growth of human lung fibroblasts [8]. Within the covRNA package, we have modified the fourthcorner and RLQ algorithms to make the methods inherently suitable for the distributional characteristics of both RNA-Sequencing (RNA-Seq) read counts and microarray intensities. We provide a parallelized high-performance implementation to make the method suitable for the analysis of large-scale multivariate gene expression data on multi-core computational systems, with additional modules for unsupervised gene filtering and plotting functions to ensure a smooth and coherent analysis workflow. Here, we demonstrate the analysis of a microarray dataset of the immune response of human dendritic cells to fungal infection [9]. In addition, in order to show the applicability of our approach to a more complex RNA-Seq data, a detailed vignette integrated in our Bioconductor package [4] demonstrates the analysis of a well-established RNA-Seq dataset of *Bacillus anthracis* [10].

### Methods

covRNA takes as input three data frames: (i) a n times m gene expression data frame L of n genes for m samples, (ii) a m times p sample annotation data frame Q of p sample covariates for m samples and (iii) a n times s gene annotation data frame R of s gene covariates for n genes. covRNA then performs a test for association between each sample and gene covariate pair following the fourthcorner procedure. Data frames R, L and Q are multiplied to yield the s times p test data frame T = R’LQ, where T_i,j_ reduces to a pairwise Pearson correlation coefficients weighted by the gene expression values of L. If both variables of a covariate pair (i,j) are categorical, the entry T_i,j_ is normalized by the sum over L to yield a Chi^2^-statistic. covRNA does not rely on any distributional assumptions as it uses a permutation test to calculate two-sided empirical p-values and makes use of Fisher’s assumption of doubling the one-sided p-value, in non-symmetric distributions [11]. Therefore, any normalization methods for microarray or RNASeq data can be used for data preprocessing. We then use permutation of the data frames to test for significant association between the covariates of R and Q. Specifically, we adopt the permutation scheme according to Ter Braak et al. [12] to ensure that all associations between gene and samples covariates are perturbed: First, the rows of L are permuted and p-values p_1_ between all covariates of R and Q are calculated. Then, the columns of L are permuted and p-values p_2_ between all covariates of R and Q are calculated. After false discovery rate correction according to Benjamini and Hochberg [13] of p_1_ and p_2_, respectively, the actual p-values are obtained by p = max(p_1_, p_2_) [12]. Taking the most conservative p-values hereby assures to model dependencies between samples and genes correctly.

The high-performance implementation of this statistical analysis in covRNA allows for straightforward parallelization on multiple available cores and significant speed-up of the analysis of large-scale datasets (Table 1).

**Table 1:** Speed-up of the fourthcorner analysis implemented in covRNA due to parallelization across multiple cores

Permutations	10^3^	10^4^	10^5^	10^6^	10^7^
1 Core (time in sec)	9.1	52.9	5.3 × 10^2^	6.8 × 10^3^	6.9 × 10^4^
10 Cores (time in sec)	8.5	15.7	84.7	7.8 × 10^2^	7.7 × 10^3^
Speed-up	1.1	3.4	6.3	8.2	9.0

The fourthcorner analysis is performed on the *Bacillus anthracis* example dataset on 1 and 10 cores for different numbers of permutations as indicated in the first row. The following rows indicate the required user time in seconds while the last row indicates the relative speed-up of the multi-threading approach. The run time was profiled on a server with 72 Cores (Intel Xeon CPU E5-2699 v3 @ 2.30 GHz) with 512 GB RAM

To visualize the relationship within and between sample and gene covariates we perform singular value decomposition on T, following the standard RLQ approach. This creates two-dimensional ordinations for both, sample and gene covariates, which are then combined into a joint ordination plot. In this plot, the covariates that are significantly associated with each other according to the statistical tests are connected by lines, whose colors reflect the type of the association (positive or negative).

### Results

We applied our method to a microarray dataset of the immune response of human dendritic cells to *Aspergillus fumigatus* (*A. fumigatus*) infection (Gene Expression Omnibus accession numbers: GSE69723, GSE77969) [9]. The ExpressionSet Expr contains gene expression data under different stimuli (‘control’, ‘LPS’ for lipopolysaccharide, ‘*A. fumigatus*’) and at different time points (‘6 h’, ‘12 h’). The genes are annotated by immune-related hallmark gene sets (n = 7 gene sets) of the MSigDB collection [3].

**Fig. 1:**
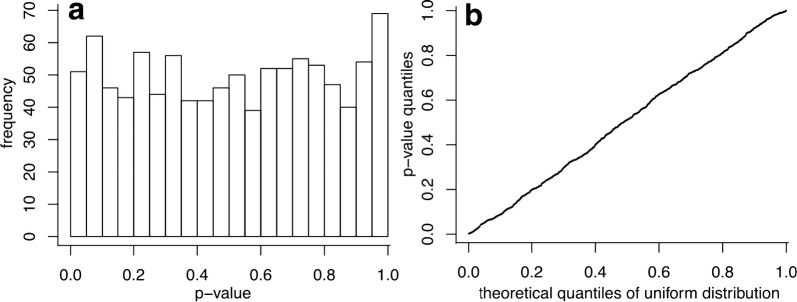
covRNA’s statistical test is shown to control the type I error rate correctly. A p-value distribution under the null hypothesis of covRNA’s statistical test between sample and gene annotations for n = 1000 permutations is generated. The results of the permutation of one random sample annotation-gene annotation association are shown here. **a** Histogram of the resulting p-values. **b** Q–Q plot of the p-values

We firstly tested if our statistical analyses were calibrated. We therefore chose an association between sample and gene annotations, and randomly permuted the gene annotation labels n = 1000 times. The resulting p-values were uniformly distributed, affirming calibration of the statistical tests (Fig. 1 for one sample annotation-gene annotation association).

**Fig. 2:**
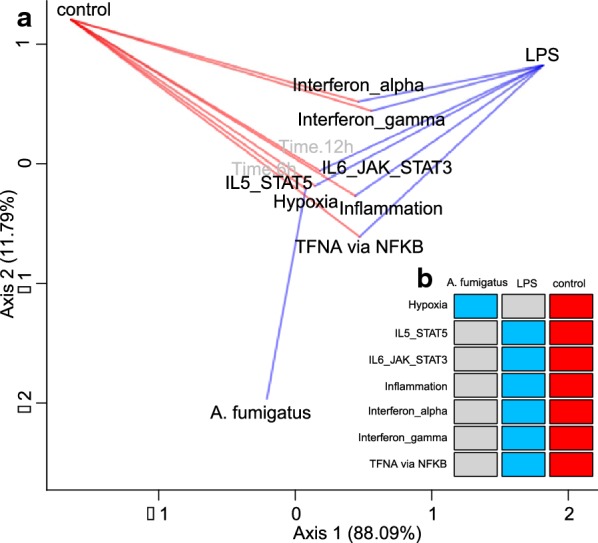
Visualization of covRNA analysis of microarray data of human dendritic cells infected with *A. fumigatus* based on the MSigDB hallmark gene set. **a** Ordination of sample and gene covariates. The lines between the covariates denote significant negative (red) and positive (blue) associations (at a significance level α = 0.05, each condition tested in turn versus the others). Gray covariates are not involved in any significant association. **b** Results of the association test. Consistently, red, blue and gray colors denote significant negative, positive or no significant associations (at a significance level α = 0.05)

Having established the calibration of covRNA’s statistical tests, we applied the covRNA methods to the microarray dataset of *A. fumigatus* infections. The following R code applied to the ExpressionSet Expr produces the results shown in Fig. 2.

*statobj *< - *stat(Expr) # statistical tests*

*ordobj *< - *ord(Expr) # ordination parameters*

*vis(statobj, ordobj) # visualization* (Fig. 2a)

*plot(statobj) # visualization of tests* (Fig. 2b)

Figure 2 illustrates the concordance of both analysis approaches. Non-associated covariates, here the two time points (6 h, 12 h) cluster around the origin of the ordination while positively/negatively associated covariates are situated at different angles from the origin (at a significance level α = 0.05; Fig. 2a). The significant associations are also summarized in a table (here n = 14 significant associations; Fig. 2b). This combined statistical and visualization analysis allows researchers to obtain a quick overview of regulatory patterns in their gene expression experiment: Here, the overview plot shows that the LPS infection of dendritic cells elicits typical bacterial infection responses like interferon activation, while a fungal infection by *A. fumigatus* leads to hypoxia in the cells. This overview confirms the successful infection of the dendritic cells in the experiment, and allows for building first hypotheses about the different molecular responses between bacterial and fungal infections.

### Discussion

The Bioconductor package covRNA provides a coherent workflow to systematically test for and visualize associations between sample and gene covariates mediated by gene expression. With only a few lines of R code, users can assess and visualize the intrinsic correlation structure of complex annotation data and discover the covariates that jointly affect the gene expression patterns. Further, experimental biologists are provided with a quick tool to validate their experiments, e.g. to assess if their stimulation assays have been successful.

The adaptation of the fourthcorner and RLQ methods, which are frequently applied in ecological landscape analyses, to the distributional characteristics of gene expression data makes the analyses accessible to a wider community. The efficient implementation and parallelization on multiple cores further allows for the analysis and visualization of large-scale multivariate gene expression datasets.

## Limitations

While one of the benefits of the covRNA package is the efficient implementation that allows scaling analyses up to thousands of genes, the analysis of too many gene and sample annotations will lead to an unclear ordination visualization with too many annotations overlapping each other. In such a case, we recommend to firstly consider the data frame visualization, to then select interesting annotations for visualization.

While covRNA tests the statistical association of annotations, it does not include a test of causality of associations. Instead, it provides a first insight into the internal structure of gene expression data.

## Data Availability

The dataset analysed in the current manuscript is available from [8]. The dataset analysed in the vignette of the Bioconductor package [1] is available from [9] and accessible via the covRNA package. Bioconductor package availablity: Project home page: https://bioconductor.org/packages/release/bioc/html/covRNA.html Operating system(s): Platform independent; multi-core systems Programming language: R License: GPL version 2 or later.

## Abbreviations

*A. fumigatus*: *Aspergillus fumigatus*
covRNA: Covariates of RNA
RNA-Seq: RNA-sequencing
